# The Etiology of Cancer

**Published:** 1898-05

**Authors:** 


### THE ETIOLOGY OF CANCER.

The cause of cancerous disease is one of the questions that has been most actively discussed for a number of years past, and that still remains without a perfectly satisfactory solution. The general medical opinion is possibly inclined to be uncertain between a provisional theory that it is a reversional tissue degeneration and one that it is of parasitic or infectious origin. The latter has had the strongest advocacy of late years in one form or another, and suspicion that it may be correct is at present, it may be, predominant.

The latest contribution to the subject is that of Dr. Roswell Park in the May issue of the *American Journal of Medical Sciences*, and like other articles from his pen, it is interesting and suggestive. According to him, in order to attack successfully this problem of the origin of cancer, "pathologists of the future must begin by studying tumor formation in the vegetable world before studying it in animals, *i. e.*, the comparative method of investigation must be adopted." He calls attention to the xylomata or woody tumors, some of which he does not hestitate to call by the name of vegetable cancers, and suggests the possibility of their being directly contagious to the human species, insects acting, it may be, as the carriers of the contagion. In support of this idea he adduces certain facts of the distribution of cancer and the researches of Noel, who found a relation between it and vegetable growths. Foresters and excise officials who have to spend much time in shady wood paths, it would seem, are especially liable to cancer, as are also those who dwell in wooded districts or shaded dwellings. This fact has not been generally recognized in the etiology of cancer, but it seems worthy of consideration and further investigation as to its universal applicability. It would be, it would seem, worth while to collect more statistics on this point and find whether malignant tumors are increasing as our prairie States become more timbered, or decrease with the clearing of the forests in those regions where deforestation is still in progress. In New York, according to the figures of the State Board of Health, quoted by Dr. Park, there has been a steady increase of deaths reported from cancer; 1,882 deaths in 1885; 2,878 in 1890, and 3,454 in 1895, or nearly twice as many as ten years before. It is not probable that the timber distribution alone could account for this difference, and so far as they go these figures give no special support to the theory suggested, but rather indicate that there must be other and still more effective causes at work that are still undiscovered. The possibility of an increasingly malignant contagion is strongly suggested by such statistics, and accords with the parasitic theories of the disorder that are coming into vogue.

Dr. Park gives an interesting review of the studies and theories of the etiology of cancer and divides them into three periods: 1, that of inaccurate observations and erroneous conclusions, beginning with Nepveau in 1872; 2, that of accurate observation, but of mistaken deduction (the coccidial period); 3, that of iconoclastic doubt, from Russell (1890) to Banti and Nisser (1894); and 4, that of successful inoculation by the Italian observers Roncali and Sanfelice within very recent years. From the results obtained by these last it would appear that the infectious nature of cancer has been fairly established, at least within certain limits, and that certain fungi, the blastomycets, are probably the most active agents of the infection. This accords with the theory of the vegetable origin of the disease, a low vegetable organism, and one of a class that has not hitherto been so commonly regarded as containing pathogenic species, being apparently the source of the infection. It is entirely possible, nevertheless, that in the future we may find yet other germs capable of exciting the morbid process under certain conditions, and among them some of those forms that have been from time to time offered as the causative factors. The failure of inoculation experiments is not an absolutely conclusive fact against their active agency, and the cancerous process in cell growths may easily be one that can be set up by more than one kind of irritation.

If cancer is increasing elsewhere at the rate it appears to be in New York State, the question of its etiology and its prevention will before long be well to the fore amongst the sanitary problems that interest our profession —*The Journal.*

